# MiR-9-5p Inhibits Glioblastoma Cells Proliferation Through Directly Targeting FOXP2 (Forkhead Box P2)

**DOI:** 10.3389/fonc.2019.01176

**Published:** 2019-11-19

**Authors:** Hongbo Zhang, Yuntao Li, Yinqiu Tan, Qi Liu, Shuting Jiang, Dongyuan Liu, Qianxue Chen, Shizhong Zhang

**Affiliations:** ^1^Department of Neurosurgery, Zhujiang Hospital, Southern Medical University, Guangzhou, China; ^2^Guangdong Provincial Key Laboratory on Brain Function Repair and Regeneration, The National Key Clinic Specialty, The Engineering Technology Research Center of Education Ministry of China, The Neurosurgery Institute of Guangdong Province, Southern Medical University, Guangzhou, China; ^3^Department of Neurosurgery, Renmin Hospital of Wuhan University, Wuhan, China; ^4^Department of Neurosurgery, Huzhou Central Hospital, Zhejiang University School of Medicine, Huzhou, China; ^5^Department of Rheumatology, The Third Affiliated Hospital of Sun Yat-sen University, Guangzhou, China

**Keywords:** miR-9-5p, FOXP2, glioblastoma, proliferation, glioma

## Abstract

Glioblastoma (GBM) is the most malignant tumor in the central nervous system and the treatment is still unsatisfactory because the mechanism of the disease remains unclear. The abnormal expression of miRNAs and its target proteins play a crucial role in the development of glioblastoma. In this study, we demonstrated that high expression of miR-9-5p and low expression of forkhead box P2 (FOXP2) were related with better outcome in patients with GBM, and down regulated FOXP2 expression was able to inhibit glioma cells proliferation by cell cycle arrest. Furthermore, we found that FOXP2 was the target protein of miR-9-5p in luciferase assay. The results of this study suggest a novel regulatory mechanism that miR-9-5p can inhibit glioma cells proliferation by downregulating FOXP2.

## Introduction

Glioblastoma (GBM) is one of the most malignant tumors in adult central nervous system. With the development of modern medical technology, it is possible to remove the tumor through surgical resection followed by chemoradiation. However, it is still a challenge to completely cure due to the strong invasive and proliferative nature of GBM. In the past decade, the prognosis and treatment for GBM have not improved significantly, but there have been important gains in our understanding on genetic alterations associated with gliomagenesis, and the gene targeting therapy might be promising for glioblastoma (1, 2).

MicroRNAs (miRNA) are a class of small non-coding RNAs and usually regulate the expression of target functional proteins through the interaction with 3′ untranslated regions (3′-UTRs) of mRNA, thus regulating the physiological and pathological processes. In the past few decades, miRNAs have been widely studied as important molecules in tumor progression, among which, the abnormal expression of miR-9 can be found in many malignancies. Interests of miR-9 have been grown with the aim of using it as a diagnostic and prognostic marker for tumors (1–3).

MiR-9 as a tumor promoter promotes the metastasis and invasion of non-small cell lung cancer (NSCLC) cells by inhibiting the expression of E-cadherin (4, 5). On the other hand, miR-9 functions as a tumor inhibitor suppressing cell proliferation and invasive ability through the SDF-1/CXCR4 pathway in epithelial ovarian cancer (6). The miR-9 can also induce cell arrest and apoptosis of oral squamous cell carcinoma via CDK 4/6 pathway (7), or down-regulate TNFAIP8 to inhibit the gastric cancer cell proliferation (8). In glioma, miR-9 inhibits glioma cells growth through various signals and promote apoptosis (9, 10). In the EGFRvIII pathway, miR-9 plays a negative role on tumorigenic capacity (11). Low expression of miR-9-3p results in a high level of Herpud1, which may protect against apoptosis in glioma (12). Over expressed miR-9 in U87 and U251 cells increases apoptosis by structural maintenance of chromosomes 1A (SMC1A) (13). It is also reported that miR-9 can reduce cell migration and the invasion of Glioblastoma cell lines through MAPK14 pathway (14). However, other researches showed different results. For example, Wu et al. found increased expression of microRNA-9 predicts an unfavorable prognosis in human Glioma (15). However, its important to note that the researches made no distinguishing between miR-9, miR-9-3p, or miR-9-5p in various tumors reported functions of miR-9.

Forkhead box P2 (FOXP2) was first found as a transcription factor involved in speech and language acquisition (16). Recently, abnormal expression of FOXP2 was found in kinds of tumors. However, the results are still controversial. Studies showed downregulation of FOXP2 in breast cancer, hepatocellular carcinoma and gastric cancer biopsies (16–18), while overexpression of FPXP2 was found in multiple myelomas, MGUS (Monoclonal Gammopathy of Undetermined Significance), several subtypes of lymphomas, osteosarcoma, neuroblastomas, and ERG fusion-negative prostate cancers (19–21).

Our previous work demonstrated miR-9-5p was a positive marker for prognosis in GBM, while FOXP2 a negative marker. FOXP2 is predicted as a direct target of miR-9-5p. In this study, miR-9-5p inhibited cell proliferation in 3 GMB cell lines *in vitro*. Reverse test was used to prove FOXP2 as a key point in miR-9-5p inhibiting GBM cells proliferation. Finally, we identified miR-9-5p inhibit tumor growth in mouse model by reducing FOXP2 expressing.

Combining these results, we conclude that miR-9-5p, through directly downregulating FOXP2 and inhibiting proliferation, is a tumor suppressor in GBM.

## Materials and Methods

### GBM Tissue Collection

All the 110 samples were collected from March 2015 to April 2017 in the Department of Neurosurgery of Renmin Hospital of Wuhan University (Wuhan, China) and Department of neurosurgery, Zhujiang Hospital of Southern Medical University (Guangzhou, China). All the tumor samples were pathologically diagnosed as glioblastoma. The study was approved by local Ethics Committee of Wuhan University and Zhujiang hospital of Southern Medical University.

### Cells and Cell Culture

Three human glioblastoma-derived cancer cell lines, U251, A118MG, and U87MG, were used in the study. The cells were purchased from the Cell Bank Type Culture Collection of Chinese Academy of Sciences, and were cultured in DMEM (Gibco) containing 10% fetal bovine serum (Gibco) and 1% penicillin-streptomycin (Sigma-Aldrich) at a temperature of 37°C and a humidified atmosphere of 5% CO_2_.

### DNA and RNA Transfection

Human FOXP2 (GenBank ID: NM_148898) full length cDNA was subcloned into a pcDNA3.1 vector to generate expression construct. All cell lines in this study were transfected with plasmids by using Lipofectamine 3000 (Thermo Fisher, USA) according to the manufacturers' protocols. FOXP2 small interfering ribonucleic acid (siRNA) (5′GACAGGCAGTTAACACTTAAT3′) and a non-specific si-RNA (as a negative control) transfections were conducted in non-serum-containing conditions using Lipofectamine 3000. All si-RNAs were used at a final concentration of 20 nM. Has-miR-9-5p mimics (5′UCUUUGGUUAUCUAGCUGUAUGA3′), negative control miRNA mimics (miR-ctrl mimics), and inhibitor (5′AGAAACCAAUAGAUCGACAUACU′3) were constructed by RiboBio (Guangzhou, China). Transfections were conducted in non-serum-containing conditions using Lipofectamine 3000 according to the manufacturer's instructions.

### Western Blot

Cell lysates were prepared by sonicating cells briefly in a modified RIPA buffer (0.1% SDS, 50 mM Tris-HCl, pH 7.5, 150 mM NaCl, 0.1% sodium deoxycholate, 1% Nonidet P-40) with proteinase and phosphatase inhibitors. BCA protein assay (TaKaRa) was used for protein quantifying. The antibody used were as follows: anti-FOXP2 (Abcam, ab16046, USA), anti-p21 (Abcam, ab109520, USA), Goat anti-Rabbit, and Goat anti-Mouse infrared dye secondary antibodies (800 CW), which were purchased from LI-COR Biosciences (Lincoln, NE, USA). Proteins were visualized with Odyssey Bioanalyzer (LI-COR).

### Real Time PCR

Total RNAs of human tissues and cells were extracted using TRIzol reagent (Invitrogen). Quantitative real-time PCR technology was used to measure the miR-9-5p expression by using the All in-One miRNA qRT-PCR Detection Kit (GeneCopoeia, Rockville,MD, USA). hsa-miR-9-5p forward primer: 5′TGCGCTCTTTGGTTATCTAGCTG3′; reverse primer: 5′CCAGTGCAGGGTCCGAGGTATT3′; U6 forward primer: 5′CGCTTCGGCAGCACATATAC 3′; reverse primer: 5′AAATATGGAACGCTTCACGA3′. All qRT-PCR processes and analyses were carried out using Applied Biosystems 7500 Fast Real-Time PCR system (Life Technologies). Relative expression of miRNA and mRNA was calculated using the 2^−ΔΔ*CT*^ method.

### Luciferase Reporter Assay

Candidate targets and its putative binding site of miR-9-5p were predicted by miRNA database (http://www.microrna.org/microrna/home.do). The 3′UTR of FOXP2, containing the wild-type or mutant miR-9-5p binding sequence, was cloned into the pMIRREPORT vector (Ambion, USA). U251 cells were cultured in 24-well plates and transfected with 0.1 μg of luciferase reporter vectors with miR-9-5p mimics or miR-ctrl mimics. The pRL-TK vector (Promega, USA) containing Renilla luciferase was also co-transfected for normalization in all experiments. Cells were harvested 48 h after transfection, and Firefly and Renilla luciferase activities were measured using the Dual-Luciferase Reporter Assay System (Promega) according to the manufacturer's protocol.

### Cell Proliferation Assay

U251, A118MG, and U87MG cell growth was measured 24, 48, and 72 h after transfection with FTL si-RNA by using the Cell Counting Kit-8 (CCK-8; Dojindo Molecular Technologies, Inc., Rockville, MD, USA), according to the manufacturer's protocol. On average, six replicates for each time point were statistically analyzed. EdU assay was also used to measure the cell growth, Cell-Light EdU Apollo488 *in vitro* Flow Cytometry Kit (20T) (RiboBio, China) was used according to the manufacturer's instructions.

### Flow Cytometry

Transfected glioma cells were trypsinized and fixed in 70% icecold ethanol at 20°C overnight. After centrifugation and wash with phosphate-buffered saline (PBS), the cells were suspended in propidium iodide (PI) working solution (50 mg/ml PI, 0.2 mg/ml RNase A, and 0.1% Triton X-100) for 30 min at 37°C. Twenty thousand cells were harvested and analyzed by FACS Calibur flow cytometry (BD Biosciences, USA).

### Tumor Formation Assay in a Nude Mouse Model

U251 cells were collected at a concentration of 2 × 10^7^ cells/mL and 0.1 ml was subcutaneously injected into either side of the armpit of male BALB/c nude mice (4–5 weeks old) the next day. Mice were purchased from Shanghai Experimental Animal Center of the Chinese Academy of Sciences (Shanghai, China). AgomiR-9-5p [micrON hsa-miR-9-5p agomiR was purchased from RiboBio (GuangZhou, China)] or agomiR control were injected into tumor at 1 nmol every 4 days for 4 times after transplanted. Tumor volumes and weights were measured every 4 days and tumor volumes were calculated using the following equation: V = 0.5 × D × d2 (V, volume; D, longest diameter; d, diameter perpendicular to the longest diameter). On the 20th day after injection, mice were killed, and the subcutaneous growth of each tumor was examined. Primary tumors were excised and tumor tissues were used to perform qPCR analysis of miR-9-5p levels. ***This study was carried out in*** ***strict accordance with the recommendations in the Guide for the Care and Use of*** ***Laboratory Animals of the National Institutes of Health. The protocol was approved by*** ***the Committee on the Ethics of Animal Experiments of the Southern Medical*** ***University*.**

### Statistical Analysis

Data were expressed as mean ± standard error. Statistical analysis was performed with SPSS 20.0 software. Differences between means were assessed by student's *t*-test for normal distribution data, or Mann–Whitney *U*-test for non-normal distribution data. In multiple comparisons, one-way analysis of variance (ANOVA) was used. Pearson's test was used to detect the correction of two groups and compare quantitative values of expression. Survival curves were plotted by the Kaplan-Meier method and compared by log-rank test. A value of *P* <0.05 was considered statistically significant.

## Results

### Expression of miR-9-5p and FOXP2 in GBM and Clinical Features

**Figure 1:**
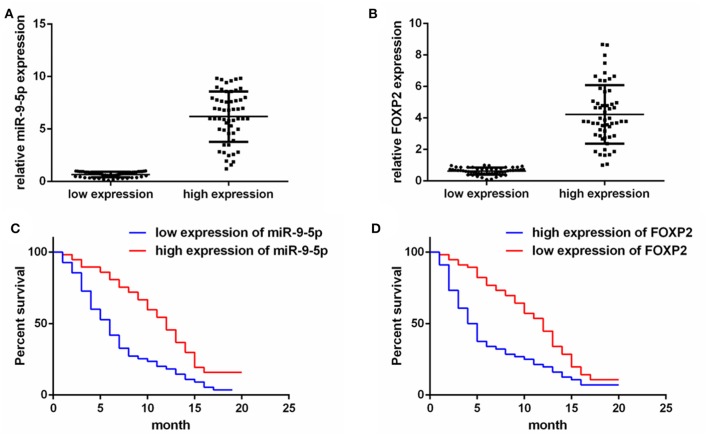
The expression of miR-9-5p and FOXP2 in glioblastoma and patients' survival. **(A)** Cases are divided into two groups according to the expression of miR-9-5p in GBM. **(B)** Cases are divided into two groups according to the expression of FOXP2 in GBM. **(C)** Kaplan-Meir survival curve analysis reveals that lower miR-9-5p predicts poorer survival (110 GBM patients). **(D)** Kaplan-Meir survival curve analysis reveals that higher FOXP2 predicts poorer survival (110 GBM patients).

To detect the expression of miR-9-5p and FOXP2 in GBM, 110 GBM samples with complete clinical and follow-up survey data were collected for this study. According to the expression level of miR-9-5p or FOXP2, cases were divided into high expression group and low expression group (Figures 1A,B). The clinical features and relative expression of miR-9-5p and FOXP2 are presented in Tables 1, 2. The cases with high expression of miR-9-5p and low expression of FOXP2 showed higher overall survival rate (Figures 1C,D).

**Table 1:** Clinical features and relative expression of miR-9 in glioblastoma (110 cases).

**Clinical variables**	**Num**	**miR-9 relative expression**		***X*^**2**^/H**	***P* value**
		**High expression**	**Low expression**		
**Total**	110	**55**	**55**		
**Gender**					
Male	51	27 (49.1)	24 (43.6)	0.329	0.566
Female	59	28 (50.9)	31 (56.9)		
**Age (y)**					
<50	48	22 (40.0)	26 (47.3)	0.591	0.442
>50	61	33 (60.0)	29 (52.7)		
**Recurrences**					
Yes	44	16 (29.1)	28 (50.9)	5.455	0.020
No	66	39 (70.9)	27 (49.1)		
**Onset time** (**m**)					
≤ 12	43	18 (32.7)	25 (45.5)	1.871	0.171
>12	67	37 (67.3)	30 (54.5)		
**Num of lobes involved**					
Single	64	40 (72.7)	24 (43.6)	9.565	0.002
Multiple	46	15 (27.3)	31 (56.4)		
**Pre-op KPS score**					
≥80	59	33 (60.0)	26 (47.3)	1.791	0.181
<80	51	22 (40.0)	29 (52.7)		
**Post-op KPS score**					
≥80	658	431 (53.4)	27 (46.6)	10.584	0.445
<80	452	124 (46.2)	328 (53.8)		
**Tumor size**					
<3 cm	35	17 (30.9)	18 (32.7)	3.251	0.197
3–5 cm	45	19 (34.5)	26 (47.3)		
>5 cm	30	19 (34.5)	11 (20.0)		
**Adjuvant therapy**					
Yes	52	23 (41.8)	29 (52.7)	1.313	0.252
No	58	32 (58.2)	26 (47.3)		
**Degree of tumor resection**					
Totally	30	12 (21.8)	18 (32.7)	0.157	0.692^a^
Most partially/partially	54	33 (60.0)	21 (38.1)		
Biopsy	26	10 (18.2)	16 (29.1)		
**Survivals**					
<6 m	37	10 (18.2)	27 (49.1)	15.209	<0.001
6–12 m	29	14 (25.5)	15 (27.3)		
>12 m	44	31 (56.4)	13 (23.6)		

*Pre-op, pre-operative; Post-op, post-operative; Num, number*.

a *Degree of tumor resection is a hierarchical variable was used by the Kruskal Wallis H-test of non-parametric test*.

**Table 2:** Clinical features and relative expression of FOXP2 in glioblastoma (110 cases).

**Clinical variables**	**Num**	**FOXP2 relative expression**		***X*^**2**^/H**	***P* value**
		**High expression**	**Low expression**		
**Total**	**110**	**55**	**55**		
**Gender**					
Male	52	27 (49.1)	25 (45.5)	0.146	0.702
Female	58	28 (50.1)	30 (54.5)		
**Age (y)**					
<50	52	28 (50.9)	24 (43.6)	0.584	0.445
>50	58	27 (49.1)	31 (56.4)		
**Recurrences**					
Yes	59	38 (69.1)	21 (38.2)	10.565	0.001
No	51	17 (30.9)	34 (61.8)		
**Onset time (m)**					
≤ 12	68	42 (76.4)	26 (47.3)	9.860	0.002
>12	42	13 (23.6)	29 (52.7)		
**Num of lobes involved**					
Single	56	32 (58.2)	24 (43.6)	2.328	0.127
Multiple	54	23 (41.8)	31 (56.4)		
**Pre-op KPS score**					
≥80	59	36 (65.5)	23 (41.8)	6.178	0.013
<80	51	19 (34.5)	32 (58.2)		
**Post-op KPS score**					
≥80	62	30 (54.5)	32 (58.2)	0.148	0.701
<80	48	25 (45.5)	23 (41.8)		
**Tumor size**					
<3 cm	34	11 (20.0)	23 (41.8)	6.972	0.031
3–5 cm	38	20 (36.4)	18 (32.7)		
>5 cm	38	24 (43.6)	14 (25.5)		
**Adjuvant therapy**					
Yes	98	50 (90.9)	48 (87.3)	0.374	0.541
No	12	5 (9.1)	7 (12.7)		
**Degree of tumor resection^b^**					
Totally	43	21 (38.2)	22 (40.0)	0.085	0.771^a^
Most partial/partial excision	43	24 (43.6)	19 (34.5)		
Biopsy	24	10 (18.2)	14 (25.5)		
**Survivals**					
<6 m	45	35 (63.6)	10 (18.2)	23.793	<0.001
6–12 m	29	10 (18.2)	19 (34.5)		
>12 m	36	10 (18.2)	26 (47.3)		

*Pre-op, pre-operative; Post-op, post-operative; Num, number*.

a *Chi-square test with continuous correction was adopted and the minimum expected value was 4.1*.

b *Degree of tumor resection is a hierarchical variable was used by the Kruskal Wallis H-test of non-parametric test*.

### miR-9-5p Was a Negative Regulator of GBM Cell Proliferation

**Figure 2:**
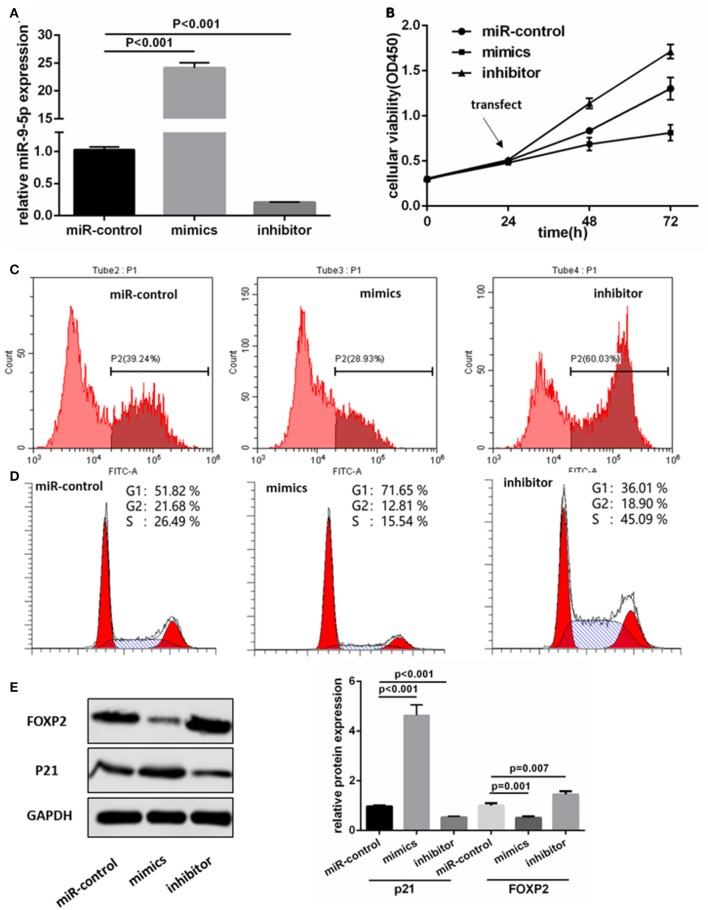
MiR-9-5p inhibits GBM cell growth through cell cycle arrest *in vitro*. **(A)** Expression of miR-9-5p in U251 cell transfected with miRNA. **(B,C)** CCK-8 **(B)** and EdU **(C)** show that high expression of miR-9-5p inhibits cell growth while low expression leads to the opposite effect. **(D)** Cell cycle is arrested in cells with high expression of miR-9-5p analyzed by Flow cytometry. **(E)** Over expression of miR-9-5p leads to down regulation of FOXP2 (*p* = 0.001) and up regulation of p21 (*p* = 0.001); while the inhibited miR-9-5p leads to up regulation of FOXP2 (*p* = 0.003) and down regulation of p21 (*p* < 0.001).

We transfected the miR-9-5p mimics, inhibitor and mi-control into the U251 and U118MG cell, and q-PCR assay showed miR-9-5p expression were significantly different (Figure 2A). CCK-8 assay was used to observe the proliferation change. Comparing with the cells transfected with mi-control, the cellular viability of the groups transfected with mimics and inhibitor was significantly decreased and increased, respectively (Figure 2B). Flow cytometry was used in EdU assay, and the results showed that less marked cells were detected in the group transfected with mimics, while more in the group with inhibitor (Figure 2C). The expression level of miR-9-5p had a significant influence on G1 phase (Figure 2D). Further, we detected proteins functioning in the cell cycle process though Western blot assay, and the expression of p21 was significantly higher in miR-9-5p (Figure 2E).

### FOXP2 Was a Positive Regulator of GBM Cell Proliferation

**Figure 3:**
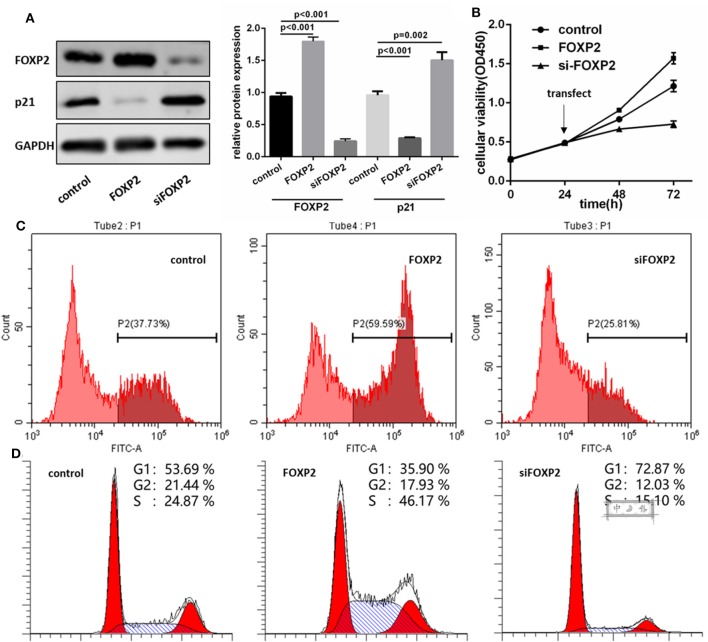
FOXP2 is a positive regulator of U87MG cell proliferation. **(A)** pcDNA3.1-FOXP2 and siRNA transfection is used to regulate the FOXP2 expression in U251 cell. Western blot shows that FOXP2 expresses as expected (*p* < 0.001). Upregulated FOXP2 leads to low expression of p21 (*p* < 0.001) and down regulated FOXP2 leads to high expression of p21 (*p* = 0.002). **(B,C)** CCK-8 **(B)** and EdU **(C)** shows high expression of FOXP2 contributes to cell proliferation while low expression leads to the opposite effect. **(D)** Cell cycle is arrested in cells with low expression of miR-9-5p analyzed by Flow cytometry.

To demonstrate that FOXP2 exerts positive effects on GBM cell proliferation, we intervened in the expression of FOXP2 (Figure 3A). Similar assays were used to analyze the cell proliferation, cell cycle and cell cycle associated proteins. Results showed that low expression of FOXP2 slowed down the cell proliferation (Figures 3B,C) and G1 arrested (Figure 3D) and inhibited p21 high expression (Figure 3A).

### miR-9-5p Directly Regulated FOXP2 Expression

**Figure 4:**
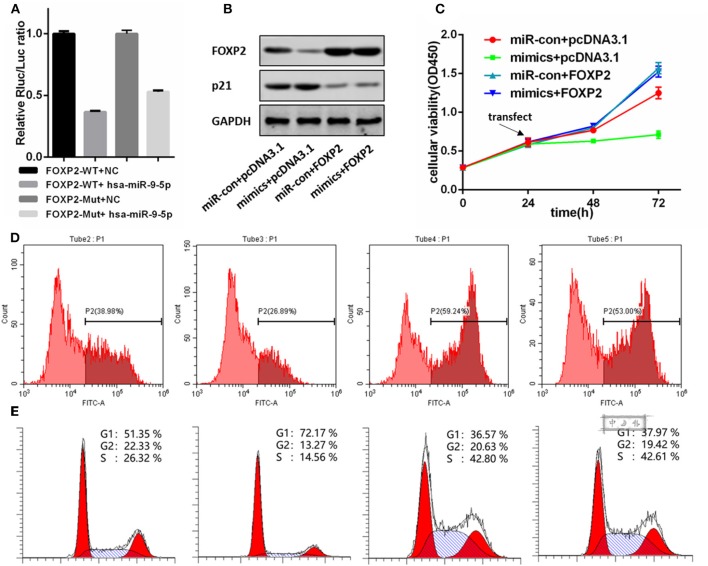
FOXP2 is indispensable for miR-9-5p suppressing U87MG cell proliferation. **(A)** Luciferase reporter assay suggests FOXP2 is the direct target of miR-9-5p. **(B)** Western bolt shows that the DNA transfection hold back the down regulation of FOXP2. Further, the up regulation of p21 caused by miR-9-5p is also restrained. **(C,D)** CCK-8 **(C)** and EdU **(D)** show that cell proliferation inhibition caused by miR-9-5p is restrained by stable expression of FOXP2. **(E)** Cell cycle arrest is blocked by the stable FOXP2.

Figure 3A shows a significant, negative relationship between the expression of FOXP2 and miR-9-5p. In GBM cells, miR-9-5p mimics resulted in FOXP2 down regulation, while the inhibitor exert a opposite effect (Figure 2E). Luciferase reporter assay suggested that FOXP2 was the direct target of miR-9-5p (Figure 4A).

### FOXP2 Was Indispensable for miR-9-5p Suppressing Tumor Growth

We re-expressed FOXP2 in the U251 cell with high level miR-9-5p by DNA transfection, and the result showed that the original cell proliferation inhibition and the cell cycle arrest caused by miR-9-5p were changed (Figures 4C–E). Further, western blot showed no difference in p21 between cells with high level miR-9-5p and the control group (Figure 4B).

### miR-9-5p Up-Regulation Inhibited GBM Growth *in vivo*

**Figure 5:**
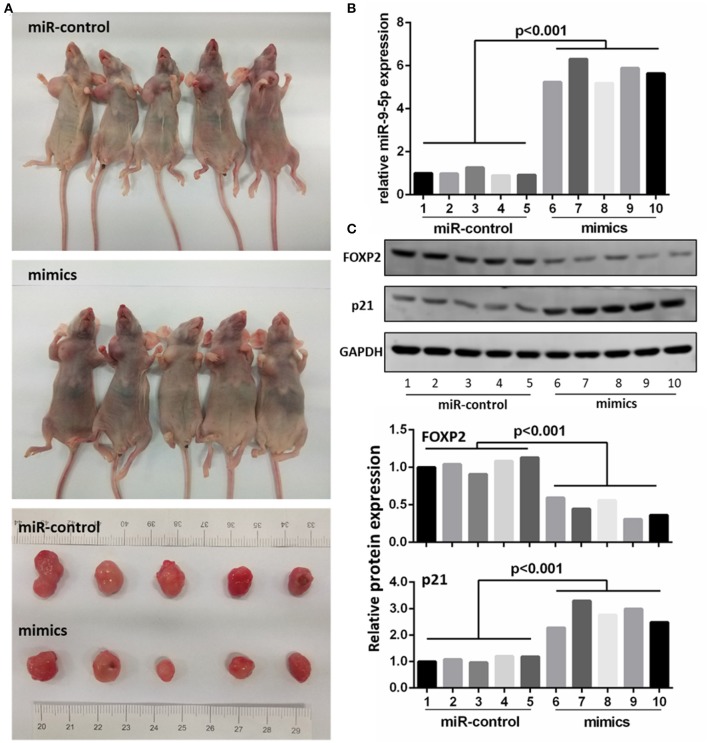
MiR-9-5p inhibits tumor growth *in vivo*. **(A)** Mice injected with miR-9-5p mimics have smaller tumors compared with control group. **(B)** q-PCR shows that miR-9-5p expression of mimics group is extremely higher than that of control group (*p* < 0.001). **(C)** Western blot shows that in mimics group, FOXP2 expression is lower (*p* < 0.001) and p21 expression is higher (*p* < 0.001).

To further investigate whether the level of miR-9-5p expression could inhibit GBM growth *in vivo*, we inoculated U251 cell into male nude mice, and injected miR-9-5p mimics or mi-control (as control) into their tail vein. Twenty-one days after injection, all mice developed xenograft tumors at the injection site and the tumor size of the mimics group was significantly smaller compared with that of the control group (Figure 5A). Moreover, the growth of tumors was significantly slower in the mimics group than that in the mi-control group (Figure 5A). The injected group showed a higher expression of miR-9-5p (Figure 5B), a lower expression of FOXP2 expression and higher p21 (Figure 5C).

## Discussion

A better understanding of key pathways driving glioma growth and development has the potential to improve the treatment of GBM. Previous studies have demonstrated that high expression of miR-9 led to lower survival rate in patients with high grade (WHO III IV) glioma (15). However, opposite results were presented in other studies. Participating in mutant EGFR signaling, the most common abnormal signaling in GBM, suppressed miR-9 expression increased GBM cells proliferation (11, 22). On the basis of the malignant behavior, Glioma is mainly divided into two types according to WHO I–IV classification (23, 24). In this study, we found that low expression of miR-9-5p led to poor prognosis while high miR-9-5p expression inhibited tumor growth by cell cycle arrest. Similar results were found in neck cancer cells (25).

For the past few years, researches have demonstrated the involvement of FOXP2 in oncogenesis (26), but it exerts opposite function in different cancers. In neuroblastomas, multiple myelomas and several subtypes of lymphomas, FOXP2 is overexpressed (19, 20, 27). In the present study, we demonstrated that FOXP2 was a tumor promoter, and it was able to accelerate the cell cycle and increase the proliferation of GBM cells. It has been reported that FOXP2 functions in neurogenesis in embryonic development (28). FOXP2 null mutant mice developed cerebellar hypoplasia (29). Consider the important effects of FOXP2 on central nervous system, we presume that once unbalanced, it may lead to GBM oncogenesis. A previous study has found that the downregulation of FOXP2 in TP53 associated glioma cell apoptosis (30), which probably indicates that FOXP2 acts as a cancer-promoting gene in GBM.

In the clinical data, FOXP2 was relatively high expressed in recurrent GBM. It is widely considered that chemotherapy resistance is a vital factor leading to GBM recurrence. FOXP2 was reported to directly target the adenosine triphosphate (ATP)-binding cassette (ABC) family proteins such as ABCA6 and ABCG2, which were transporters expelling chemical compounds (31–33). This might be a mechanism in chemotherapy resistance of GBM and a reason for why relatively high expression of FOXP2 was found in recurrent GBM.

## Conclusion

In this study, we confirmed that the high expression of miR-9-5p, which down regulated FOXP2, was able to suppress the proliferation of GBM via p21-dependent cell cycle arrest both *in vivo* and *in vitro*. It suggests a potential pathway of miR-9-5p-FOXP2 signal which may be applied to GBM therapy in the future.

## Data Availability Statement

The datasets during and/or analyzed during the current study are available from the corresponding author on reasonable request.

## Ethics Statement

This study was carried out in strict accordance with the recommendations in the Guide for the Care and Use of Laboratory Animals of the National Institutes of Health. The protocol was approved by the Committee on the Ethics of Animal Experiments of the Southern Medical University.

## Author Contributions

HZ and YL planned and designed the experimental scheme and the initial draft of the paper, tumor sample collection, and performed experiment. YT, QL, SJ, and DL participated in the cell culture, microRNA extraction experiments, and participated in the collation of experimental data, statistics, and results analysis. QC and SZ were fully responsible for the implementation and supervision of the subject.

### Conflict of Interest

The authors declare that the research was conducted in the absence of any commercial or financial relationships that could be construed as a potential conflict of interest.
